# Eicosanoyl-5-hydroxytryptamide (EHT) prevents Alzheimer’s disease-related cognitive and electrophysiological impairments in mice exposed to elevated concentrations of oligomeric beta-amyloid

**DOI:** 10.1371/journal.pone.0189413

**Published:** 2017-12-18

**Authors:** Kesava Asam, Agnieszka Staniszewski, Hong Zhang, Scott L. Melideo, Adolfo Mazzeo, Michael Voronkov, Kristen L. Huber, Eduardo Pérez, Maxwell Stock, Jeffry B. Stock, Ottavio Arancio, Russell E. Nicholls

**Affiliations:** 1 Department of Pathology and Cell Biology, Columbia University, New York, NY, United States of America; 2 The Taub Institute for Research on Alzheimer’s Disease and the Aging Brain, Columbia University, New York, NY, United States of America; 3 Department of Molecular Biology, Princeton University, Princeton, New Jersey, United States of America; 4 Signum Biosciences, 133 Wall Street, Princeton, New Jersey, United States of America; 5 Department of Medicine, Columbia University, New York, NY, United States of America; Nathan S Kline Institute, UNITED STATES

## Abstract

Soluble forms of oligomeric beta-amyloid (Aβ) are thought to play a central role in Alzheimer’s disease (AD). Transgenic manipulation of methylation of the serine/threonine protein phosphatase, PP2A, was recently shown to alter the sensitivity of mice to AD-related impairments resulting from acute exposure to elevated levels of Aβ. In addition, eicosanoyl-5-hydroxytryptamide (EHT), a naturally occurring component from coffee beans that modulates PP2A methylation, was shown to confer therapeutic benefits in rodent models of AD and Parkinson’s disease. Here, we tested the hypothesis that EHT protects animals from the pathological effects of exposure to elevated levels of soluble oligomeric Aβ. We treated mice with EHT-containing food at two different doses and assessed the sensitivity of these animals to Aβ-induced behavioral and electrophysiological impairments. We found that EHT administration protected animals from Aβ-induced cognitive impairments in both a radial-arm water maze and contextual fear conditioning task. We also found that both chronic and acute EHT administration prevented Aβ-induced impairments in long-term potentiation. These data add to the accumulating evidence suggesting that interventions with pharmacological agents, such as EHT, that target PP2A activity may be therapeutically beneficial for AD and other neurological conditions.

## Introduction

Alzheimer’s disease (AD) is a debilitating neurodegenerative condition for which no effective disease modifying treatment exists. AD is currently the 6^th^ leading cause of death in the United States [1], and the prospect that its incidence will increase as the population ages makes it a source of increasing public health concern. We found previously that eicosanoyl-5-hydroxytryptamide (EHT), a component of coffee, showed therapeutic benefits in rodent models of AD and Parkinson’s disease [2–4]. Here we examine further the effect of EHT on AD pathogenesis and show that EHT administration reduces the sensitivity of mice to behavioral and electrophysiological impairments caused by the AD-linked protein, beta-amyloid (Aβ).

EHT was identified in a screen for natural compounds that enhance the activity of the serine/threonine protein phosphatase, PP2A, toward phospho-protein substrates associated with AD and PD [3]. Multiple lines of evidence independently implicate PP2A in AD, among these are the observations that 1) PP2A expression and activity are reduced in brains from AD patients [5–8], 2) reducing PP2A activity in animal models results in AD-like pathology and cognitive deficits [9–15], 3) PP2A is the principal phosphatase for phosphorylated forms of tau linked to AD [16], and 4) pharmacological activation of PP2A reduces cognitive impairment and pathology in mouse tauopathy models [17–19].

PP2A is a heterotrimeric protein composed of a catalytic subunit (C), a structural subunit (A), and a regulatory subunit (B). Multiple isoforms exist for each of these subunits and they are assembled in a highly-regulated process [20]. The identity of the B subunit is thought to be the principal determinant of substrate specificity, with B55α subunit-containing enzymes exhibiting the highest tau phosphatase activity [21]. The proportion of PP2A heterotrimers that contain B55α subunits is regulated by methylation and demethylation of the catalytic subunit catalyzed by a dedicated methyltransferase and methylesterase, in a process that is conserved from yeast to mammals [20]. Dysregulated PP2A activity resulting from impaired PP2A methylation is thought to be one of the molecular mechanisms by which hyperhomocysteinemia leads to increased AD risk [22].

Aβ is a 40–42 amino acid peptide generated by proteolytic cleavage of the amyloid precursor protein (APP). Aβ is the primary constituent of the amyloid plaques that characterize AD, and soluble forms of Aβ produce AD-related impairments in cell and animal models [23]. To examine the effect of dysregulated PP2A methylation on AD pathogenesis, we previously generated two lines of transgenic mice that overexpress either the PP2A methyltransferase, LCMT- 1, or the PP2A methylesterase, PME-1. In published work, we showed that PME-1 over expression sensitized animals to cognitive and electrophysiological impairments caused by Aβ exposure, while LCMT-1 overexpression protected animals from these impairments [24]. In the current study, we tested whether EHT might also protect mice from the pathological actions of Aβ. We found that EHT treatment decreases sensitivity to Aβ-induced cognitive and electrophysiological impairments in a manner similar to LCMT-1 over expression. These data add to the accumulating evidence suggesting that PP2A, or enzymes that regulate it, may constitute viable therapeutic targets for AD prevention or treatment.

## Results

### Effect of EHT on Aβ-induced cognitive impairments

To test the effect of EHT on Aβ-induced cognitive impairments, we shifted 3-month-old wild-type mice to diets containing either 0, 0.01 or 0.1% EHT [3] 3 weeks prior to surgery to implant bilateral cannulae directed at the dorsal hippocampus. These doses of orally administered EHT were shown previously to protect against impairments resulting from the expression of a virally-transduced PP2A inhibitor in rats [2], as well as Parkinson’s disease-related impairments in α-synuclein expressing transgenic mice, and mice injected with MPTP [3, 4]. In the most recent of these studies, EHT treatment showed efficacy against MPTP-induced impairments after only 4 weeks of administration, so this was selected as the minimum time for *in vivo* EHT administration in the current experiments. After 7–10 days of recovery from surgery, animals were then tested on a battery of behavioral tasks conducted over the course of 2 weeks in the following order: open field behavior, 2-day radial arm water maze, contextual fear conditioning, visible platform water maze and sensory threshold assessment. Prior to testing in each task, animals were infused with 1 μl per side of 200 nM synthetic oligomeric Aβ peptide or vehicle as described in [24]. Oligomeric Aβ was prepared from synthetic peptide corresponding to the human Aβ 1–42 sequence and oligomerized according to a previously described protocol [25]. In earlier studies, administration of synthetic oligomeric Aβ prepared in this manner showed a hormetic dose-response relationship with respect to both its electrophysiological and behavioral effects [26, 27], and the 200 nM *in vivo* dose selected for these studies corresponds to the threshold at which maximal cognitive impairment was observed [27].

**Fig 1:**
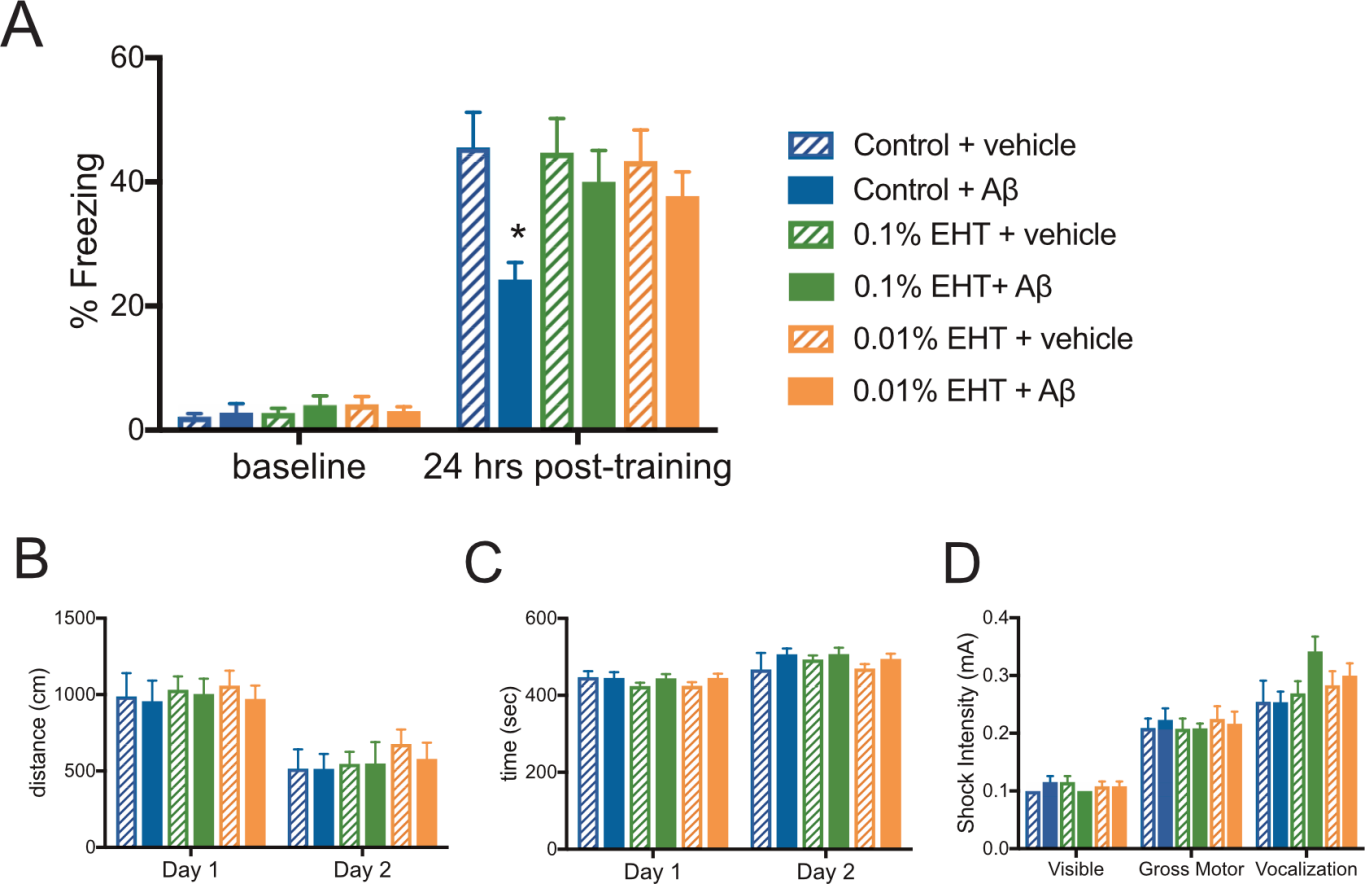
EHT prevents Aβ-induced impairment of contextual fear conditioning. **(**A) Average percent of time spent freezing (± SEM) during initial exposure to the training context (baseline) and 24 hours after foot shock for the indicated treatment groups. 2-way RM-ANOVA with group and training day as factors: F(5,77) = 2.749, P = 0.0244 for group; F(1,77) = 300.5, P<0.0001 for training day, and F(5,77) = 2.301, P = 0.0528 for interaction, Bonferroni post-hoc comparisons of the control + vehicle group to all other treatment groups in the experiment show that only the control + Aβ group is significantly different than control + vehicle group at 24 hrs, P = 0.0002. No significant differences were observed between groups in their baseline responses. (N = 13 control + vehicle, 13 control + Aβ, 14 0.01% EHT + vehicle, 14 0.01% + Aβ, 14 0.1% + vehicle, 15 0.1% EHT + Aβ.) **(**B) Average distance traveled (± SEM) for the indicated treatment groups during 10 min exposures to an open field environment on subsequent days. 2-way RM-ANOVA with group and day as factors: F(5,68) = 0.3755, P = 0.8647 for group; F(1,68) = 45.31, P<0.0001 for training day, and F(5,68) = 0.0702, P = 0.9965 for interaction. (C) Average time immobile (± SEM) for the indicated treatment groups during 10 min exposures to an open field environment on subsequent days revealed no significant differences between groups 2-way RM-ANOVA with group and day as factors: F(5,68) = 0.8474, P = 0.5187 for group; F(1,68) = 24.7, P<0.0001 for training day, and F(5,68) = 0.5231, P = 0.7585 for interaction. (N = 11–14 per group). (D) Plot of average threshold for responses to foot shocks of increasing intensity for the indicated treatment groups 2-way RM-ANOVA with group and threshold as factors: F(5,67) = 0.7218, P = 0.6094 for group; F(2,134) = 158.2, P<0.0001 for threshold, and F(10,134) = 1.548, P = 0.1291 for interaction. (N = 11–14 per group).

We found that animals treated with diets containing either 0.01 or 0.1% EHT were resistant to Aβ-induced impairments in a contextual fear conditioning task (Fig 1A). This task requires animals to make an association between an aversive foot shock, and a novel context, and has been found to be both hippocampus-dependent and sensitive to elevated Aβ levels [24, 28]. Hippocampus-dependent memory impairments are also a prominent feature of AD. All animals were infused with vehicle or Aβ 20 min prior to their first exposure to the conditioning chamber, and no significant differences in baseline freezing were observed among these groups prior to foot shock administration. Upon reintroduction to the conditioning chamber 24 hrs later, all vehicle-treated groups showed similarly elevated freezing responses indicative of strong memories for the shock-context association, while animals on control diet infused with Aβ showed reduced freezing responses compared to controls, suggesting that Aβ infusion interfered with these memories. Notably, Aβ-infused animals on EHT-containing diets exhibited freezing responses that were comparable to vehicle infused controls, suggesting that EHT administration protected animals from the cognitive impairment caused by Aβ administration.

To test for possible differences in baseline activity levels among these groups that might confound our interpretation of their behavior in the contextual fear condition task, we examined their behavior in an open field environment. To test for possible differences in shock perception among these groups that might affect their performance in the contextual fear condition task, we also examined their behavioral responses to a range of shock intensities. We found that neither dietary EHT administration nor Aβ infusion 20 min prior to testing affected their ambulatory activity, as assessed by total distance travelled (Fig 1B) and immobility time (Fig 1C). We also found comparable thresholds for the first visible, first gross motor, and first vocal response to foot shocks of increasing intensity among these groups (Fig 1D). Together these data suggest that the differences observed among these groups during testing in the contextual fear conditioning task are not due to differences in baseline activity levels or shock perception.

**Fig 2:**
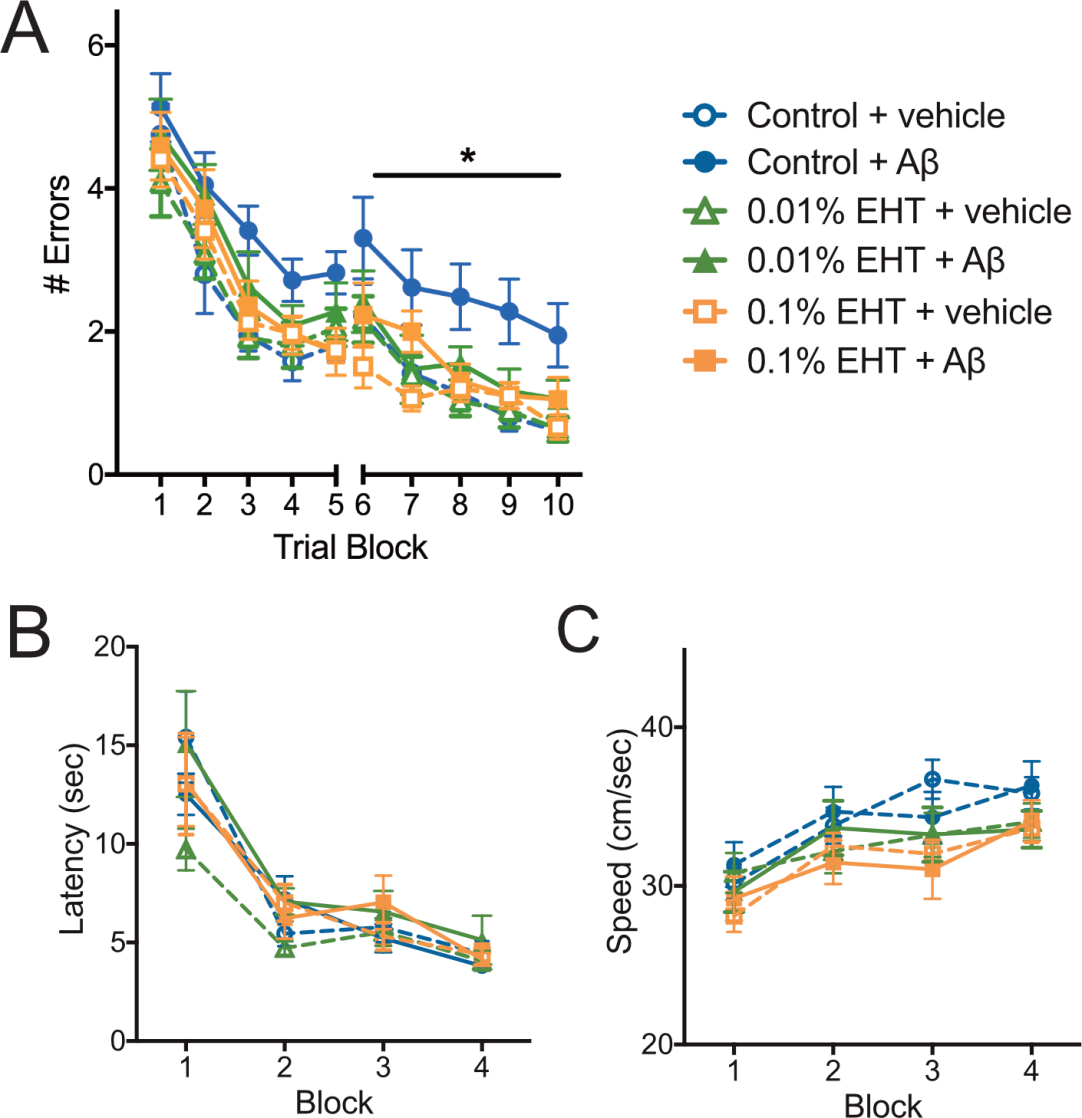
EHT prevents Aβ-induced impairment of spatial learning and memory in a 2-day radial arm water maze task. **(**A) Average number of errors committed (± SEM) during each 3-trial training block of a 2-day radial arm water maze task for the indicated treatment groups. 2-way RM-ANOVA for day 2 (blocks 6–10) with block and group as factors: F (5,69) = 4.424, P = 0.0015 for group; F (4,276) = 25.95, P<0.0001 for block,; F (20,276) = 0.5657, P = 0.9338 for interaction. Bonferroni post-hoc comparisons of the control + vehicle group to all other treatment groups show that only the control + Aβ group is significantly different than control + vehicle group. (N = 12 control + vehicle, 13 control + Aβ, 12 0.01% EHT + vehicle, 12 0.01% + Aβ, 13 0.1% + vehicle, 13 0.1% EHT + Aβ.) (B) Plot of the average escape latency (± SEM) for the indicated treatment groups during training on a visible platform Morris water maze task reveals no significant differences between groups (2-way RM-ANOVA with trial block and treatment group as factors: F(5,69) = 0.9766, P = 0.4384 for group, F(3,207) = 72.48, P<0.0001 for block, and F(15,207) = 0.8627, P = 0.6068 for interaction). (N = 12 control + vehicle, 13 control + Aβ, 12 0.01% EHT + vehicle, 12 0.01% + Aβ, 13 0.1% + vehicle, 13 0.1% EHT + Aβ). (C) Plot of the average swim speed (± SEM) for the indicated treatment groups during training on the visible platform Morris water maze task described in B reveals no significant differences between groups (2-way RM-ANOVA with trial block and treatment group as factors: F(5,69) = 1.232, P = 0.3035 for group, F(3,207) = 28.3, P<0.0001 for block, and F(15,207) = 0.9227, P = 0.5398 for interaction).

As an additional test of EHT’s ability to protect against Aβ-induced cognitive impairments, we tested these animals on 2-day radial arm water maze task. This task is a test of short-term spatial memory and requires animals to learn and remember the identity of visual cues to navigate to a specific location. It is also hippocampus-dependent and sensitive to elevated Aβ levels [29]. We found that all vehicle treated groups acquired this task at a comparable rate across trials, and to a comparable extent (Fig 2A). As described previously [24], administration of 200 nM Aβ 20 min before and again midway through training on each day of the task resulted in impaired performance in animals fed the control diet. However, Aβ administration to animals on EHT-containing diets did not cause similar impairments, and the performance of the Aβ-infused, EHT-treated groups was similar to that of the vehicle-infused control group on this task. Like the data from the contextual fear conditioning task, these results suggest that EHT administration protects against cognitive impairments caused by elevated Aβ levels.

To test for potential differences in visual perception, motivation or swimming ability among these groups that might affect their performance in the 2-day radial arm water maze task, we assessed their performance in a visible platform version of the Morris water maze. We found no significant differences among these groups in either escape latency (Fig 2B), or swimming speed (Fig 2C) during 4 3-trial training blocks carried out across 2 days, suggesting that differences in these variables do not account for the differences observed among groups in the 2-day radial arm water maze task.

### Effect of EHT on Aβ-induced impairments in synaptic plasticity

**Fig 3:**
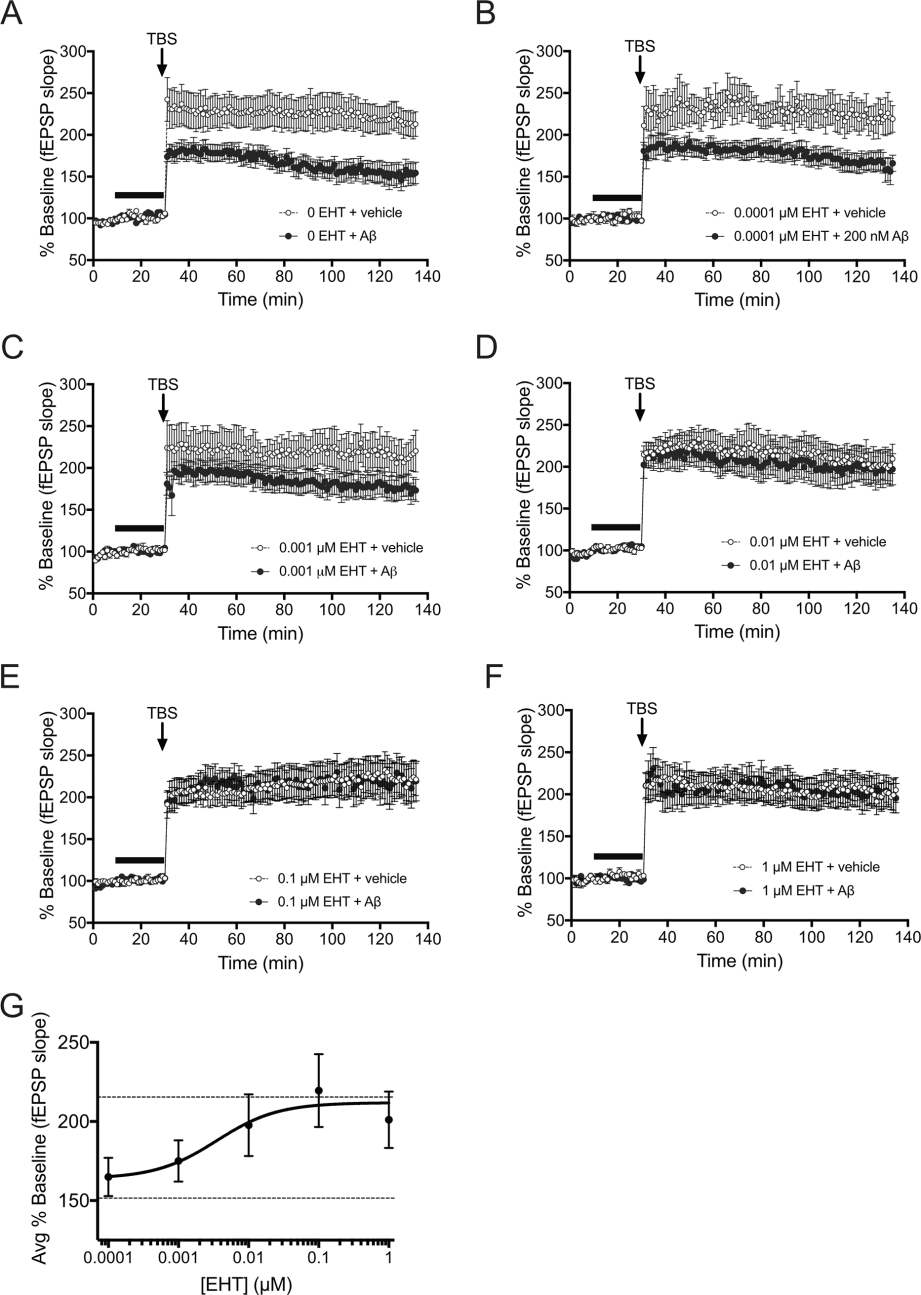
Acute EHT treatment prevents Aβ-induced impairment of long-term potentiation. **(**A-F) Time course of averaged Schaffer collateral fEPSP responses (± SEM) in hippocampal slices prepared from slices treated with vehicle or 0, 0.0001, 0.001, 0.01, 0.1, or 1 μM EHT +/- vehicle or 100 nM Aβ (horizontal bar) 20 min prior to delivery of theta-burst stimulation (arrow). (A) Aβ treatment significantly reduces potentiated responses following TBS in slices treated with 0 μM EHT (2-way RM-ANOVA for treatment with time and treatment as factors: F(1,19) = 8.827, P = 0.0078). (B) Aβ treatment significantly reduces potentiated responses following TBS in slices treated with 0.0001 μM EHT (2-way RM-ANOVA for treatment with time and treatment as factors: F(1,11) = 6.84, P = 0.0240). (B) Aβ treatment yields a non-significant trend for reduced potentiated responses following TBS in slices treated with 0.001 μM EHT (2-way RM-ANOVA for treatment with time and treatment as factors: F(1,16) = 2.123, P = 0.1645). (D-F) Aβ treatment does not significantly reduce potentiated responses following TBS in slices treated with 0.01, 0.1, or 1 μM EHT (2-way RM-ANOVA for treatment with time and treatment as factors: For 0.01 μM EHT: F(1,18) = 0.1646, P = 0.6898; For 0.1 μM EHT: F(1.21) = 0.0034, P = 0.9543; For 1 μM EHT: F(1,15) = 0.0014, P = 0.9702). Comparison of potentiated responses in the absence of Aβ revealed no effect of EHT treatment alone on TBS-induced LTP (2-way RM-ANOVA comparisons to 0 EHT + vehicle for treatment with time and treatment as factors: For 0.0001 μM EHT: F(1,15) = 0.0132, P = 0.9099; For 0.001 μM EHT: F(1,18) = 0.0539, P = 0.8191; For 0.01 μM EHT: F(1,18) = 0.16, P = 0.6939; For 0.1 μM EHT: F(1,21) = 0.2294, P = 0.6369; For 1 μM EHT: F(1,18) = 0.7098, P = 0.4106). (G) Plot of the average potentiated responses over the last 10 min of the recordings shown in B-F for slices treated with Aβ in the presence of the indicated concentrations of EHT. The upper and lower dashed lines indicate the mean potentiated response obtained in the absence of EHT for vehicle or Aβ treated slices respectively. (N = 11 0 μM EHT + vehicle, 10 0 μM EHT + Aβ, 6 0.0001 μM EHT + vehicle, 7 0.0001 μM EHT + Aβ, 9 0.0001 μM EHT + vehicle, 9 0.0001 μM EHT + Aβ, 9 0.01 μM EHT + vehicle, 11 0.01 μM EHT + Aβ, 12 0.1 μM EHT + vehicle, 11 0.1 μM EHT + Aβ, 9 1 μM EHT + vehicle, 8 1 μM EHT + Aβ slices).

Activity-dependent changes in the efficacy of synaptic transmission within the hippocampus are thought to be required for particular forms of learning and memory, and interference with these changes, caused by elevated levels of Aβ is thought to contribute to AD-associated cognitive impairments [30]. To determine whether EHT administration also affected Aβ-induced impairments in synaptic plasticity, we measured long-term potentiation (LTP) in the presence or absence of Aβ in hippocampal slice preparations treated acutely with EHT (Fig 3A–3F). EHT was bath applied at 0, 0.0001, 0.001, 0.01, 0.1, or 1 μM together with 100 nM Aβ or vehicle for 20 min prior to administration of a theta-burst stimulus train (TBS). As in the behavioral tests, the dose of Aβ selected for in vitro application in this experiment was at or near the previously reported threshold for maximal LTP impairment in these preparations [26, 27]. In the absence of EHT, TBS resulted in potentiated responses that were significantly reduced by pretreatment with Aβ (Fig 3A). However, this impairment was prevented by EHT in a dose-dependent manner (Fig 3), with slices treated with 0.0001 μM EHT still showing a significant impairment (Fig 3B), slices treated with 0.001 μM EHT showing an intermediate impairment (Fig 3C), and slices treated with 0.01μM EHT or higher, showing no significant Aβ-induced LTP impairment (Fig 3D–3F). Analysis of the average potentiated responses for the last 10 min of these recordings yield an EC50 for EHT-mediated protection against Aβ-induced LTP impairments of approximately 3.5 nM (Fig 3G). In addition, the magnitude of LTP obtained in EHT treated slices in the absence of Aβ was similar to LTP obtained in control slices treated with neither EHT nor Aβ, suggesting that LTP was not affected by EHT treatment at the indicated concentrations (Fig 3A–3F). Interestingly, the efficacy of EHT in these assays was significantly greater than the micromolar efficacy for EHT-mediated inhibition of PP2A demethylation reported previously [3], and, while significant differences may exist between the *in vitro* and *in vivo* dose-response relationships for EHT’s protective effects, they are also consistent with the higher efficacy of 0.01% EHT-containing diets in protecting against Aβ-induced vs. α-synuclein-related impairments in that study.

**Fig 4:**
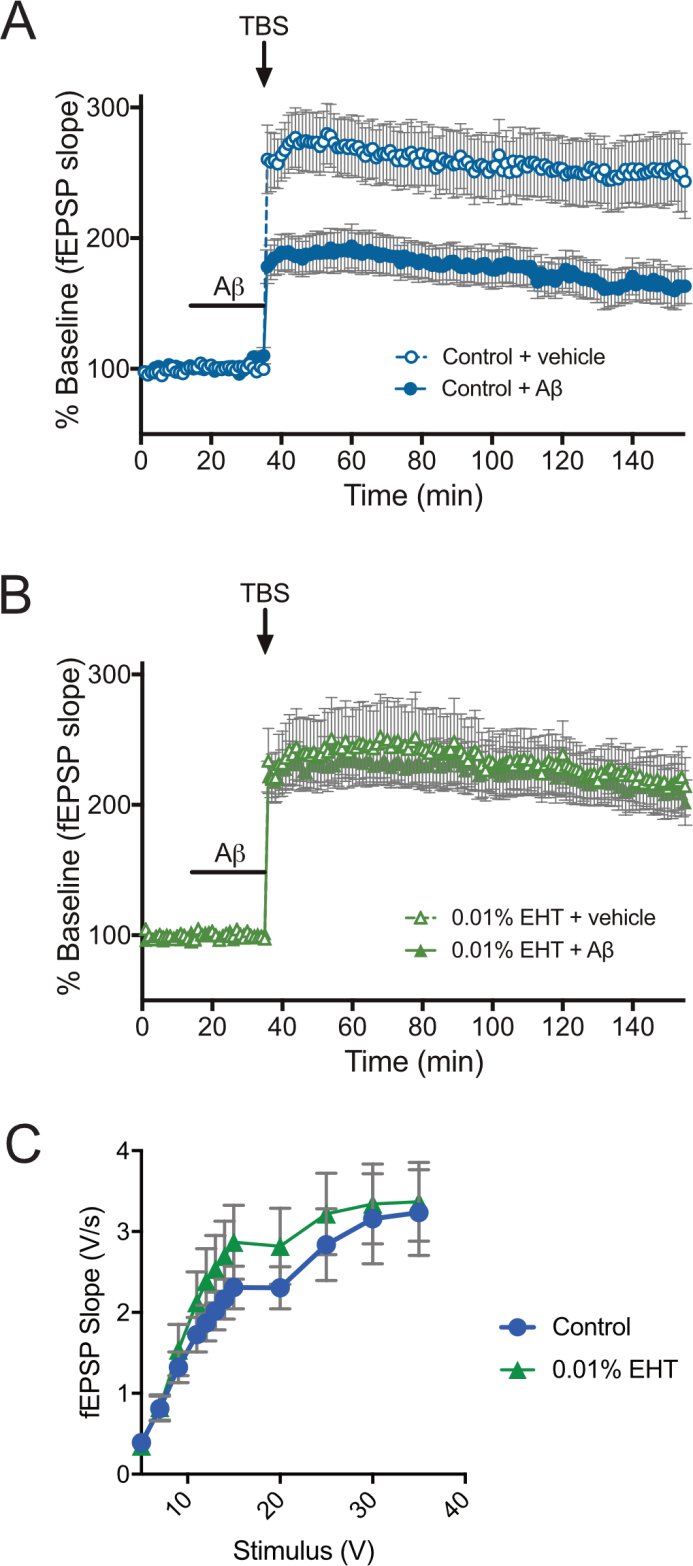
Chronic EHT treatment prevents Aβ-induced impairment of long-term potentiation. **(**A-B) Time course of averaged Schaffer collateral fEPSP responses (± SEM) in hippocampal slices prepared from animals fed control or EHT-containing diets and treated with either vehicle or 100 nM Aβ (horizontal bar) 20 min prior to delivery of theta-burst stimulation (arrow). (A) Aβ treatment significantly reduces potentiated responses following TBS in slices prepared from animals on control diets (2-way RM-ANOVA for treatment with time and treatment as factors: F(1,29) = 8.913, P = 0.0057). (B) Mice fed diets containing 0.01% EHT are resistant to Aβ-induced LTP impairment (2-way RM-ANOVA for treatment with time and treatment as factors: F(1,26) = 0.0943, P = 0.7612). C) Input/output (2-way RM-ANOVA for treatment with stimulus and treatment as factors: F(1,57) = 0.5466, P = 0.4628) (N = 31 control, 28 0.01% EHT).

To determine whether chronically administered EHT might also protect against Aβ-induced LTP impairments *in vivo*, we prepared acute hippocampal slices from the same vehicle-infused EHT treated animals used in the behavioral assays. We then performed extracellular field potential recordings of LTP at Schaffer collateral synapses in these preparations in the presence or absence of 100 nM Aβ. As expected, TBS produced robust potentiation of responses in vehicle-treated slices prepared from animals that received control diet that were significantly reduced by Aβ treatment (Fig 4A). In contrast, Aβ treatment did not significantly affect potentiated responses in slices from animals that received diets containing 0.01% EHT (Fig 4B)–consistent with the hypothesis that EHT treatment may protect against Aβ-induced cognitive impairments by preventing underlying Aβ-induced impairments in synaptic plasticity. To test for possible effects of EHT treatment on baseline synaptic transmission, we compared the input/output relationships at Schaffer collateral synapses in slices prepared from animals that received 0 or 0.01% EHT containing diets, and found no significant differences among these groups (Fig 4C).

### Effect of EHT treatment on PP2A expression and substrate phosphorylation

**Fig 5:**
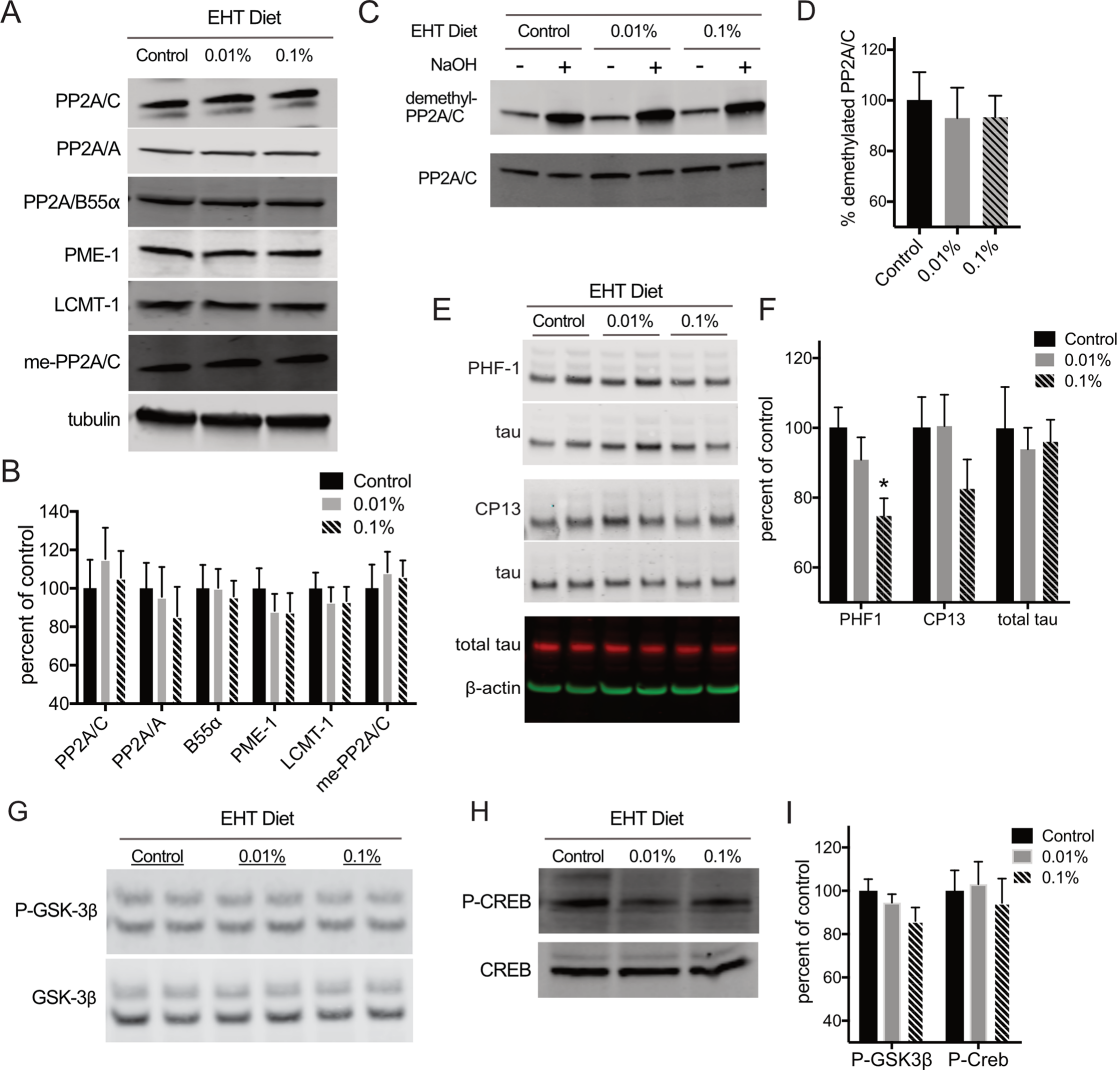
EHT treatment reduces tau phosphorylation. **(**A) Representative western blots for the indicated proteins and methylated PP2A/C performed on hippocampal homogenates prepared from animals fed control diet, or diets containing 0.01 or 0.1% EHT. (B) Histogram showing average ± SEM for tubulin-normalized band intensities expressed as average percent of control band intensity from replicate western blots in A show no significant differences in expression levels for any of the indicated proteins or for methylated PP2A/C (ANOVA for: PP2A/C: F(2,22) = 0.2533, P = 0.7784; PP2A/A: F(2,22) = 0.2588, P = 0.7743; B55α: F(2,22) = 0.06221, P = 0.9399; PME-1: F(2,22) = 0.4942, P = 0.6167; LCMT-1: F(2,22) = 0.2498, P = 0.7812; methyl-PP2A/C: F(2,22) = 0.1666, P = 0.8476). (C) Representative western blots for demethylated PP2A, and total PP2A/C performed on the homogenates described in A either treated (+) or mock treated (-) with 0.5 M sodium hydroxide. (D) Histogram of average demethylated PP2A/C (± SEM) in hippocampal homogenates prepared from animals fed control, or 0.01 or 0.1% EHT containing-diets show no significant differences in demethylated PP2A/C levels (ANOVA: F(2,22) = 0.1436, P = 0.8670). Values were calculated as ratios of demethyl-PP2A/C to total PP2A/C band intensities for -NaOH treated samples from replicate western blots shown in C and expressed as percent of the average of control. (E) Representative western blots performed on hippocampal homogenates prepared from animals fed control diet, or diets containing 0.01 or 0.1% EHT for phospho-Ser396/404 (PHF1), phospho-Ser202 (CP13) together with their corresponding total tau loading controls, as well as and total tau together with its corresponding β-actin loading control. (F) Histogram showing average band intensities ± SEM for phospho-Ser396/404-tau (PHF1), phospho-Ser202-tau (CP13) normalized to corresponding total tau loading control, and total tau normalized to corresponding β-actin loading control for replicate western blots shown in H show a trend for reduced phosphorylation at these sites in EHT-treated animals (ANOVA for: PHF1: F(2,22) = 5.147, P = 0.147, Bonferroni post-hoc for PHF1 0.1% EHT vs. Control: t = 3.154, P = 0.0092; CP13: F(2,22) = 1.433, P = 0.2599; total tau: F(2,22) = 0.1268, P = 0.8815). (G and H) Representative western blots for phospho-Ser9 and total GSK3B and phospho-Ser133 and total Creb performed on hippocampal homogenates prepared from animals fed control diet, or diets containing 0.01 or 0.1% EHT. (I) Histogram showing average ± SEM of phospho-GSK3β and phospho-Creb band intensities normalized to corresponding total GSK3β and Creb respectively for replicate western blots shown in G&H show no significant effect of EHT treatment on phosphorylation at these sites (ANOVA for: P-GSK3β F(2,22) = 1.761, P = 0.1952; P-Creb: F(2,22) = 0.1776, P = 0.8385). (N = 8 control, 8 0.01% EHT and 9 0.1% EHT treated animals for each measure).

To probe the molecular mechanisms underlying the effect of EHT treatment on Aβ sensitivity, we compared the levels of PP2A and some of its associated proteins and post-translational modifications in 3–4 month-old wild-type mice treated for 4 weeks with diets containing 0, 0.01, or 0.1% EHT. We found no significant differences between controls and animals maintained on EHT-containing diets for PP2A/C or PP2A/A subunit expression or in the level of the B55α subunit expression whose incorporation into PP2A holoenzymes is thought to be regulated by C-subunit methylation (Fig 5A and 5B). We also did not observe changes in the expression of the PP2A methylesterase, PME-1, or the PP2A methyltransferase, LCMT-1, suggesting that EHT’s effects on Aβ sensitivity were not due to alterations in the levels of these PP2A subunits or the enzymes that regulate PP2A methylation.

At micromolar concentrations, EHT acts as an inhibitor of PME, and this is likely one mechanism by which EHT increases PP2A activity when administered *in vivo* [3]. To examine whether EHT-related changes in PP2A methylation might underlie its effects on Aβ sensitivity, we used antibodies selective for the methylated (Fig 5A and 5B), and demethylated forms of PP2A/C (Fig 5C and 5D) to compare the level of PP2A/C methylation in animals maintained on control and EHT-containing diets. While prolonged dietary treatment with EHT from weaning results in detectable changes in the level of PP2A/C subunit methylation in healthy wild type mice and rats [2, 3], short-term treatment does not elicit similar changes [4], and we observed no significant differences in PP2A/C subunit methylation between the EHT-treated and control groups in our experiments (Fig 5B and 5D). This result is consistent with the normal behavioral performance we observe in EHT-treated animals that were not exposed to Aβ, and with the observation that PP2A/C methylation levels are already nearly saturated under basal conditions in mouse brain, reducing the likelihood of detecting any additional increase in methylation resulting from EHT administration (e.g. the dimethyl-PP2A/C signal in -NaOH treated control samples in Fig 5C is 21.9 ± 2.5% of the corresponding value in +NaOH treated control samples). This result is also consistent with our previous work on transgenic mice that over express LCMT-1, where we did not detect a significant increase in PP2A methylation under basal conditions despite significantly reduced Aβ sensitivity in these animals [24].

To explore the possible mechanisms by which EHT affects Aβ sensitivity in these animals, we compared the phosphorylation levels of three PP2A substrates that have been implicated in AD: tau, GSK3β, and Creb. Tau has been suggested to act downstream of, or at least cooperatively with Aβ in pathways leading to AD-related behavioral and electrophysiological impairments [31]. Since PP2A is the principal tau phosphatase in the brain, decreased tau phosphorylation, through increased PP2A activity, is one mechanism by which EHT administration might alter sensitivity to Aβ-induced impairments. To explore this possibility, we examined tau phosphorylation at two phospho-epitopes using the PHF-1 antibody that recognizes tau phosphorylated on serines 396 or 404 [32], and the CP13 antibody that recognizes tau phosphorylated on serine 202 [33]. We found that EHT at the highest concentration tested significantly reduced PHF1 immunoreactivity under basal conditions, with a trend toward reduction at the CP13 epitope that was not statistically significant (Fig 5E and 5F).

GSK3β is another PP2A substrate that has been implicated in AD [34]. Dephosphorylation of GSK3β at serine 9 resulting from increased PP2A activity could lead to an increase in tau phosphorylation mediated by the active form of this kinase. However, recent data suggest dephosphorylation of GSK3β is not mediated by methylation-sensitive PP2A isoforms [35]. To determine whether EHT administration leads to changes in GSK3β phosphorylation that might impact Aβ induced impairments or tau phosphorylation, we compared the level of GSK3β phosphorylation at serine 9 by western blot using a phospho-specific antibody. We found that EHT treatment resulted in no significant change in GSK3β phosphorylation (Fig 5G and 5I), however the trend revealed in these data suggest that EHT-dependent changes in GSK3β activity could potentially compete with its effects on PP2A-mediated tau dephosphorylation.

The cAMP responsive transcription factor, Creb, is key mediator of pathways required for memory that has also been implicated in AD [36]. Creb is activated through phosphorylation on serine 133, and can be dephosphorylated at this site by both PP2A and PP1 [37]. Creb dephosphorylation has been associated with a decrease in synaptic efficacy which could potentially contribute to AD related cognitive impairments [23, 38]. To determine if EHT administration impacts Creb phosphorylation, we compared the level of phospho-serine 133 imunoreactivity in animals receiving 0, 0.01, or 0.1% EHT and found no significant differences among these groups (Fig 5G and 5H), suggesting that EHT administration does not affect Creb-mediated synaptic plasticity under basal conditions.

## Discussion

Here we report that administration of EHT prevents cognitive and electrophysiological impairments caused by acute application of oligomeric Aβ in mice. Given the central role that oligomeric Aβ is thought to play in AD pathogenesis, these results suggest that EHT administration may represent an effective therapeutic strategy for preventing AD-related impairments that result from elevated levels of Aβ. These data are consistent with our published results showing that promoting PP2A methylation by transgenic overexpression of the PP2A methyltransferase, LCMT-1, also protected mice from Aβ-induced cognitive and electrophysiological impairments [24]. Our current data also show that EHT administration did not affect baseline performance in any of the behavioral tasks tested, or TBS-induced LTP suggesting that, like LCMT-1 overexpression, EHT may selectively block the pathological effects of Aβ without impairing its normal physiological function. The protective effects of EHT shown here are also consistent with our published results showing that dietary EHT administration prevented AD-related pathology in rats that express a PP2A-inhibiting transgene [2]. However, since multiple beta-amyloid species have been identified in AD brains [39, 40], we cannot rule out the existence of Aβ species not present in our synthetic preparations that lead to impairments in individuals with AD via mechanisms that are insensitive to EHT administration.

Here, we tested the effects of EHT on Aβ sensitivity at EHT doses that were found previously to protect against impairments resulting from PP2A inhibition in rats, and transgenic α-synuclein expression and MPTP injection in mice [2–4]. In one of those studies, we found evidence for a dose-dependent relationship between EHT and Parkinson’s disease-related impairments in mice that express an α-synuclein transgene [3]. However, in the current experiments, we found that diets containing 0.01% and 0.1% EHT were equally effective in protecting against Aβ-induced impairments in our assays. Moreover, we found that chronic dietary and acute *in vitro* administration of EHT were both effective in protecting against Aβ-induced LTP impairments, and only at 0.1 nM did we observe a loss of efficacy for acutely administered EHT in our LTP experiments. The concentrations of Aβ used in these experiments were near the threshold for maximal behavioral and electrophysiological impairments, and this could account for a quantitative difference in the amount of EHT necessary to protect against Aβ-induced impairments in these assays compared with earlier results obtained using different experimental paradigms. Alternatively, these data could suggest a qualitative difference in the sensitivity of the pathways that mediate the pathological actions of α-synuclein and Aβ to EHT, with Aβ-induced impairments potentially being more sensitive to EHT administration.

EHT was initially identified as a compound that activates PP2A by inhibiting the PP2A methylesterase, PME-1 [3], and the effects of EHT treatment in these and previous experiments are consistent with a PP2A-dependent mechanism of action. However, as in our previous study of LCMT-1 over expressing transgenic mice [24], we were unable to detect significant changes in PP2A methylation in these EHT-treated animals despite profound decreases in Aβ sensitivity. This may be due to the high percentage of methylated PP2A/C subunits that exist under basal conditions, as well as the duration of treatment (since treatment with EHT from weaning in previous studies elicited detectable changes PP2A/C methylation [2, 3]). However, we cannot rule out that EHT may be affecting Aβ sensitivity in these experiments via a mechanism other than PME-1 inhibition.

PP2A is thought to be the principal phosphatase for phosphorylated forms of tau that are linked to AD [41], and tau has been found to affect AD-related impairments that result from elevated levels of Aβ. A mechanism by which PP2A mediated increases in tau dephosphorylation underlie the effects of EHT on Aβ-induced impairments is therefore an appealing explanation for the results obtained in the current experiments. Our observation that EHT treatment reduces basal levels of tau phosphorylation in animals that were not exposed to Aβ is consistent with this mechanism of action (Fig 5E and 5F). In fact, since Aβ exposure is thought to lead to increased tau phosphorylation [42–46], the effects of EHT may be more pronounced in animals exposed to elevated Aβ levels. While our current data are not inconsistent with a role for tau dephosphorylation in EHT-dependent decreases in Aβ sensitivity, the number of PP2A isoforms and substrates and the complexity of PP2A regulation leave open the possibility that other PP2A substrates may play a role in mediating EHT’s neuroprotective effects. Nevertheless, the results of the current experiments add strong support to the accumulating evidence suggesting that agents such as EHT that modulate PP2A activity may provide useful therapeutics for multiple neurological disorders including AD [2, 24] and other tauopathies [17, 18], as well as impairments resulting from traumatic brain injury [47–52], and Parkinson’s disease [3, 4].

## Materials and methods

### Animals

Equal numbers of 3–4 month-old male and female wild type F1 mice generated from crosses of C57BL6/J and 129SVEV/Tac animals were used for all experiments. Animals were housed individually following surgeries to implant cannulae. Surgical procedures were performed under anesthesia and all efforts were made to minimize suffering. Behavioral testing was conducted during the light phase of a 12 light/dark cycle. Animals were tested in cohorts of 10–12 animals consisting of equal numbers of each of the 6 Aβ/EHT treatment groups. Behavioral testing consisted a battery of tasks carried out over a period of 2 weeks in the following order: open field behavior, 2-day radial arm water maze, contextual fear conditioning, visible platform water maze and sensory threshold assessment. All procedures involving animals were conducted in strict accordance with protocols approved by the Columbia University Institutional Animal Care and Use Committee (USDA Registration #21-R-0082; AAALAC Accreditation #000687; NYDOH #A141).

### Pharmacological agents

EHT was prepared and synthesized at Signum Biosciences as described in [53]. For acute treatments, EHT was dissolved in dimethyl sulfoxide at a concentration of 1 mM and stored in aliquots at -20°C until use. Final dilutions were prepared in DMSO such that a volume of 40 μl of EHT diluted in DMSO was added to 40 ml of artificial cerebral spinal fluid (ACSF) to achieve the final concentrations described above. Vehicle treatments were performed by adding of DMSO only. EHT containing and control diets were prepared by Research Diets (New Brunswick, NJ) as described in [3]. Diets containing either 0, 0.01 or 0.1% EHT were provided *ad libidum* as described in the text. Synthetic Aβ peptide corresponding to amino acids 1–42 of human amyloid precursor protein was purchased from the UCLA Biopolymer Laboratory (Los Angeles, CA) and oligomerized according to previously described protocols [25, 54] (S1 Fig).

### Cannulation and Aβ infusion

For experiments involving *in vivo* infusion of Aβ, animals were implanted with a 26-gauge guide cannula (Plastics One, Roanoke, VA) into the dorsal part of the hippocampi (coordinates: P = 2.46 mm, L = 1.50 mm to a depth of 1.30 mm) [55] under anaesthesia with 20 mg/kg Avertin. Cannulas were fixed to the skull with acrylic dental cement (Paladur) and animals were allowed to recover for 6–8 days following surgery prior to behavioural testing. 1 μl of Aβ was infused into each hippocampus at a concentration of 200 nM over a period of 1 minute through cannulas connected to a microsyringe by a polyethylene tubing at the timepoints indicated in the text. After infusion, the needle was kept in place for an additional minute to allow diffusion of Aβ into the tissue.

### Open field behavior

Animals were placed into a plexiglass chamber (27.3 cm long × 27.3 cm wide × 20.3 cm high) for 10 min on each of two successive days during which time their movements were tracked using a arrays of infrared beams and a computerized tracking system and analyzed using behavioral analysis software (Med Associates).

### Radial arm water maze

Testing was performed in a 120 cm diameter pool containing a six arm radial maze insert and filled with opaque water as described previously [29]. Mice were tested in 15 x 1 minute trials on each of 2 consecutive days. The location of the escape platform was held constant during testing but the start location was pseudorandomly varied throughout. On the first day, training alternated between visible and hidden platform trials, while on the second day only hidden platform trials were conducted. Water temperature was maintained at approximately 24°C and mice were dried and placed in a clean heated cage between trials to prevent hypothermia. Entries into maze arms that did not contain the escape platform were scored as errors. Data are presented as the average number of errors committed during blocks of 3 training trials.

### Contextual fear conditioning

Animals were placed into a conditioning chamber (33cm x 20cm x 22cm) made of transparent Plexiglas on two sides and metal on the other two located inside a sound-attenuating box (72cm x 51cm x 48cm) (MED Associates, St Albans, VT). Background white noise (72dB), was provided through a speaker installed in one of the side of the sound-conditioning chamber and illumination was provided by a 24 W bulb. A clear Plexiglas window allowed the experimenter to record and monitor freezing behavior using a video camera connected to a personal computer. Foot shocks were administered through a removable 36-bar grid floor and the entire apparatus was cleaned and deodorized between animals with distilled water and 70% ethanol. Animals were placed in the conditioning chamber once on each of two consecutive days. On the first day of exposure mice were placed in the conditioning chamber for 2 minutes before the onset of a discrete 30s, 2800Hz, 85dB tone, the last 2s of which coincided with a 0.5 mA foot shock. After the tone and shock exposure, the mice were left in the conditioning chamber for another 30s before returning to their home cages. 24 hours after their first exposure, animals were returned to the conditioning chamber for 5 min without foot shock or tone presentation. Freezing behavior during all phases of testing was calculated using FreezeFrame software (Med Associates).

### Visible platform water maze

This task was conducted in the same 120 cm diameter pool used for the radial arm water maze task but with the partitions removed. Training for this task was carried out over 2 days with 3 morning and 3 afternoon trials on each day. Intertrial intervals were 15 to 20 min and rest periods between morning and afternoon sessions were 2–3 hrs. Each trial was a maximum of 120 sec during which time the animals were required to swim to a visible escape platform located just above the water surface. Animals that did not reach the platform within the allotted time were guided to it and allowed to sit there for 15 sec before returning to their home cage. The location of the platform was varied among 4 different locations such that it was not present in the same location on any two successive trials. Water temperature was maintained at approximately 24°C, and animals were dried and placed in a clean warmed cage after each trial to prevent hypothermia. Animal movements were recorded using a video-tracking system and time required to reach the hidden platform (latency) and swim speed were determined using Ethovision behavioral analysis software (Noldus).

### Sensory threshold assessment

Animals were placed into the same apparatus used for contextual fear conditioning. A sequence of single, 1sec foot shocks were then administered at 30 sec intervals and 0.1 mA increments from 0 up to a maximum of 0.7 mA. Each animal’s behavior was monitored by the experimenter to determine their thresholds for first visible response to the shock (flinch), their first gross motor response (run/jump), and their first vocalized response.

### Electrophysiological studies

Extracellular field potential recordings were performed on acute hippocampal slices prepared as described previously [54] from wild-type animals that received control and EHT containing diets. Animals were euthanized by cervical dislocation—a method of euthanasia approved by the Panel on Euthanasia of the American Veterinary Medical Association that yields viable anesthetic-free tissue suitable for electrophysiological recordings. Brains were then rapidly removed and cooled in ice cold ASCF consisting of in mM: 124 NaCl, 4.4 KCl, 1 Na_2_HPO_4_, 25 NaHCO_3_, 2 CaCl_2_, 2 MgCl_2_, and 10 glucose. Hippocampi were the dissected and sliced into 400 μM sections using a tissue chopper. Slices were incubated at 29°C in an interface chamber under continuous perfusion (2 ml/min) with oxygenated ACSF and allowed to recover for a minimum of 90 min prior to recording responses in the CA1 region to stimulation of Schaffer collateral projections with a bipolar electrode. Input/output relationships were determined prior to each recording and stimulus intensities that elicited 30% of the maximal response were utilized. Stable baselines were obtained for a minimum of 15 min prior to drug or vehicle application and a theta-burst stimulation protocol consisting of 3 trains separated by 15 second intervals with each train consisting of 10 bursts at 5 Hz and each burst consisting of 5 pulses at 100 Hz was used to elicit LTP.

### Western blotting

After 4 weeks of dietary EHT administration animals were euthanized by cervical dislocation–an AVMA approved method that allows for rapid removal of non-hypoxic brains. Hippocampi were then rapidly dissected, snap frozen and stored prior to homogenization for western blot analysis. Hippocampal homogenates were prepared by sonication at 95°C in aqueous buffer containing 2% lithium dodecyl sulfate and 50 mM Tris pH 7.5. Total protein concentrations were determined by bicinchoninic acid assay according to the manufacturer’s instructions (Pierce) and 15 μg of total protein was loaded per lane on SDS-PAGE gels. Proteins were transferred to PVDF membranes, which were then blocked with Odyssey Blocking Buff (TBS) (LI-COR) for 1 hr at rm temp. Blots were probed with primary antibodies (Table 1) overnight followed by the corresponding secondary, which was one of the follow: Goat anti-rabbit (IRDye 800CW LI-COR), Goat anti-mouse (IRDye 680RD LI-COR), or Donkey anti-goat (IRDye 680RD LI-COR). Protein bands were detected by an Odyssey Clx and quantified by ImageStudio. Band intensities were determined for each antigen and normalized to the corresponding within-lane loading control. Data were presented as the mean normalized band intensity for each treatment group ±SEM and expressed as the percent of the mean value for the vehicle-treated control group.

**Table 1:** List of antibodies.

Protein	Cataolg/Company	Dilution
LCMT-1	ab119320/Abcam	1:1,000
PME-1	07–095 EMD/Millipore	1:2000
Tubulin	MAB3408/Millipore	1:5,000
N-terminus PP2Ac (Total PP2Ac)	Sc-130237/Santa Cruz	1:200
Methylated PP2Ac	4D9/ Princeton University [57]	1:500
Demethylated PP2Ac	1D6/Millipore	1:1000
PP2A subunit A	07-250/Millipore	1:1000
GSK-3β	Ab2602/Abcam	1:1000
GSK-3β-P (Ser9)	9336/Cell Signaling	1:1000
CREB	Sc-186/Santa Cruz	1:100
CREB-P (Ser133)	Sc-7978/Santa Cruz	1:200
PP2A subunit B55-α	Sc-81606/Santa Cruz	1:200
phospho-tau S396/404	PHF-1Peter Davies	1:500
phospho-tau S202	CP13/Peter Davies	1:500
Total tau	PA5-27287/Pierce	1:5,000
β-actin	MA5-15739/Pierce	1:5,000
Aβ	803004 (6E10)/Biolegend	1:1000

### Alkaline demethylation

To demethylate PP2Ac, the following method was modified from Yu et al. [56]. Homogenates were incubated with 0.5 M NaOH for 10 min at 4°C. The reaction was stopped by first adding HCl at a final concentration of 0.460 mM and then Tris-HCl at a final concentration of 0.1 M.

## Supporting information

S1 Fig Oligomeric Aβ preparation. Representative western blot of synthetic Aβ preparation used in behavioral and electrophysiological assays. Synthetic peptide corresponding to human Aβ 1–42 sequence was incubated for 24 hrs at 4°C in artificial cerebral spinal fluid. The incubated preparation was then separated by SDS-PAGE on a 10% NuPAGE bis-Tris gel, and probed with the monoclonal antibody 6E10 which recognizes an epitope contained within residues 1–17. The pattern of immunoreactivity observed is consistent with that originally reported in Stine et al, [25] and in subsequent publications [58, 59]. It should be noted, however, that while these data show that the oligomeric Aβ preparations used in this study were similar in this assay to preparations used in earlier studies, they do not necessarily provide an accurate representation of the oligomeric state of these preparations in aqueous solution [60].
(TIFF)
