# Supplementary figures and images for: Eicosanoyl-5-hydroxytryptamide (EHT) prevents Alzheimer’s disease-related cognitive and electrophysiological impairments in mice exposed to elevated concentrations of oligomeric beta-amyloid
Source: PLoS One. 2017 Dec 18;12(12):e0189413. doi: 10.1371/journal.pone.0189413 (PMC5734769; doi:10.1371/journal.pone.0189413)

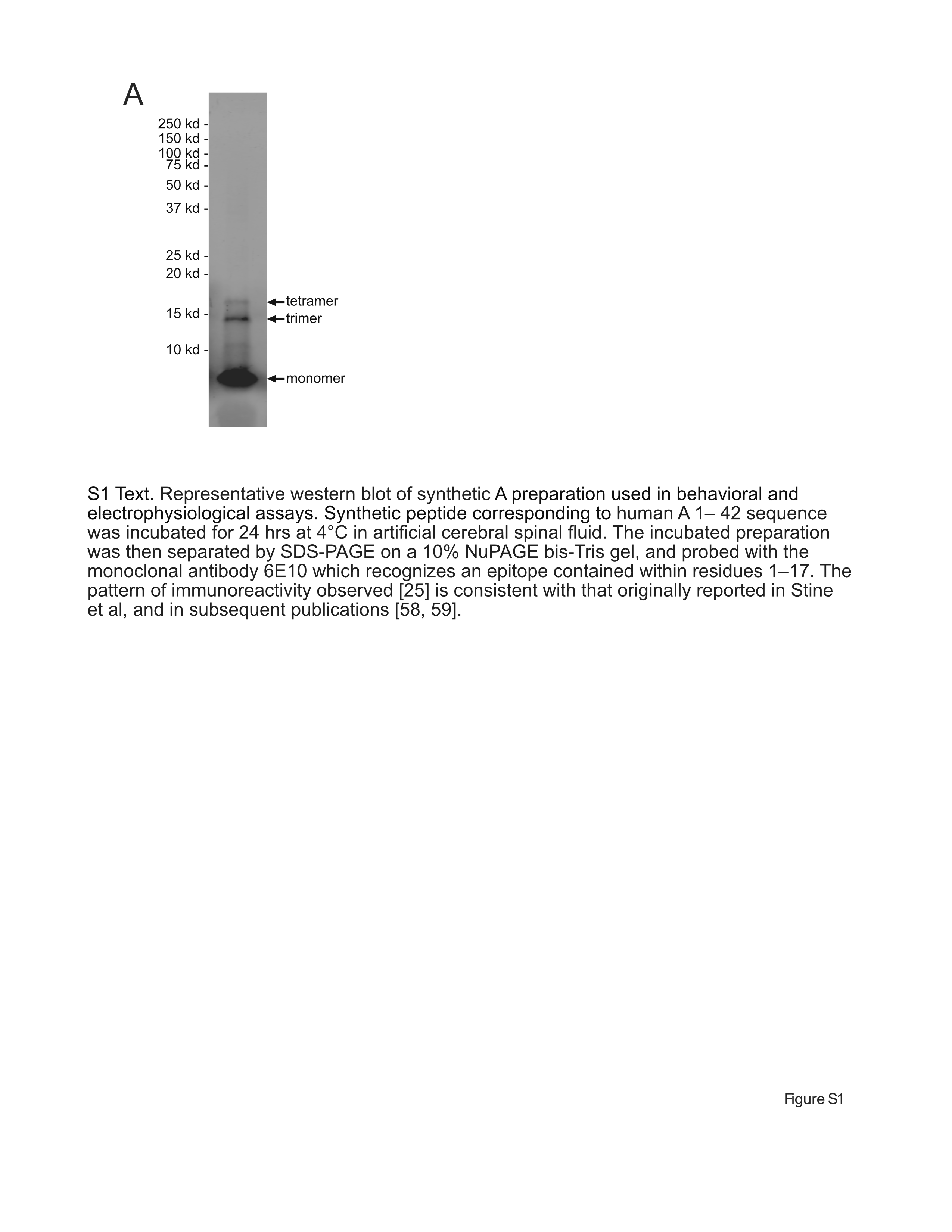

Supplement: S1 Fig — Representative western blot of synthetic Aβ preparation used in behavioral and electrophysiological assays. Synthetic peptide corresponding to human Aβ 1–42 sequence was incubated for 24 hrs at 4°C in artificial cerebral spinal fluid. The incubated preparation was then separated by SDS-PAGE on a 10% NuPAGE bis-Tris gel, and probed with the monoclonal antibody 6E10 which recognizes an epitope contained within residues 1–17. The pattern of immunoreactivity observed is consistent with that originally reported in Stine et al, [25] and in subsequent publications [58, 59]. It should be noted, however, that while these data show that the oligomeric Aβ preparations used in this study were similar in this assay to preparations used in earlier studies, they do not necessarily provide an accurate representation of the oligomeric state of these preparations in aqueous solution [60]. (TIFF) [file pone.0189413.s001.tiff]
